# Supplementary material for: N-Terminal Acetyltransferase Naa40p Whereabouts Put into N-Terminal Proteoform Perspective
Source: Int J Mol Sci. 2021 Apr 1;22(7):3690. doi: 10.3390/ijms22073690 (PMC8037211; doi:10.3390/ijms22073690)
Supplement: Supplementary file 1 [file ijms-22-03690-s001.zip › Figure_S1.pdf]

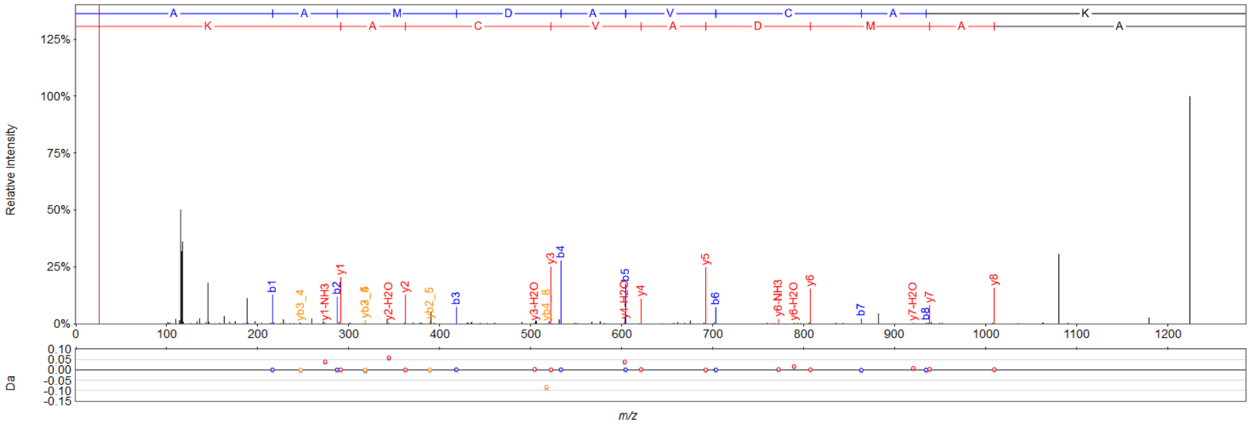

Figure S1| Representative MS^2 spectrum matching a doubly charged precursor mass of *m/z* 935.4205, corresponding to the modified peptide sequence AAMDAVC<Cmm>AK matching AA-sequence 20 to 28 of hNaa40p as reported in ProteomicsDB ([1], accessed January 2021) and with ‘Cmm’ and ‘iTRAQ’ denoting an carbamidomethyl and iTRAQ moiety.

Reference

1. Samaras, P. et al. (2020) ProteomicsDB: a multi-omics and multi-organism resource for life science research. Nucleic Acids Res 48 (D1), D1153-D1163.
